# Editorial: Glucose metabolism in brain and neurodegenerative diseases: multifactorial effects

**DOI:** 10.3389/fnagi.2026.1991548

**Published:** 2026-10-26

**Authors:** Rita Machado de Oliveira, Ivan Nalvarte, Andrea González

**Affiliations:** 1 NOVA Medical School, Faculdade de Ciências Médicas (NMS, FCM), Universidade NOVA de Lisboa, Lisbon, Portugal; 2 iNOVA4Health, NOVA Medical School, Faculdade de Ciências Médicas (NMS, FCM), Universidade NOVA de Lisboa, Lisbon, Portugal; 3 Department of Neurobiology, Care Sciences and Society, Division of Neurogeriatrics, Karolinska Institutet, Solna, Sweden; 4 Faculty of Veterinary Medicine and Agronomy, Institute of Natural Science, University of the Americas, Santiago, Chile

**Keywords:** Brain glucose metabolism, metabolic dysfunction, insulin signaling, Alzheimer's disease, neurodegenerative diseases, glial metabolism, GLP-1 receptor agonists, precision metabolic medicine

The brain is one of the most metabolically active organs in the body, consuming approximately 20% of total energy despite accounting for only about 2% of body mass (Magistretti and Allaman, 2015). This high demand reflects the continuous need for energy to sustain neuronal activity, synaptic transmission, and cognition. Disturbances in cerebral glucose metabolism can disrupt brain homeostasis and are associated with neurodegenerative disease.

Over the past two decades, epidemiological, clinical, and experimental studies have linked obesity, insulin resistance and type 2 diabetes with neurodegeneration. Impaired insulin signaling and glucose metabolism, mitochondrial dysfunction, oxidative stress, neuroinflammation, and vascular dysfunction may form an interconnected network linking systemic metabolic dysfunction to brain pathology. However, the direction and possible contribution of these pathways remain poorly understood.

This Research Topic was launched to provide a platform for studies examining links between glucose metabolism and neurodegenerative diseases including AD (González et al., 2022), Parkinson's disease (PD)(Chen et al., 2025), Huntington's disease (HD) (Morea et al., 2017) and frontotemporal dementia (FTD) (Joshy et al., 2025). Candidate mechanisms include altered insulin signaling (Bosco et al., 2011; Kellar and Craft, 2020), neuroinflammation, and hormonal regulation of brain metabolism, although their relative contribution and strength of evidence differ across disorders. When the topic was launched, diabetes and obesity were established risk factors for neurodegeneration, whereas metabolic pathways as therapeutic targets were still emerging. The collection is predominantly focused on AD, with one contribution on cerebral small vessel disease, and reflects both the complexity of these interactions and the progress of the field.

## Insulin signaling and glucose metabolism in brain homeostasis

A first theme is the role of insulin signaling in brain homeostasis. The review on glucagon-like peptide-1 (GLP-1) receptor signaling, Li et al., examines links between diabetes and AD, and discusses incretin signaling as a potential strategy to target metabolic and neuronal dysfunction simultaneously. The review by Su et al. on insulin resistance in cerebral small vessel disease, extends the discussion beyond AD, linking impaired insulin signaling with vascular pathology, cerebrovascular dysfunction, and cognitive impairment. Together, these studies support highlight the relevance of insulin signaling accross neurodegenerative and cerebrovascular disease.

## Cellular crosstalk shapes cerebral energy metabolism

A second theme concerns the complexity of cerebral energy metabolism itself, which depends on coordinated interactions between neurons, astrocytes, oligodendrocytes, endothelial cells, and immune cells, not neuronal glucose uptake alone. The review on glial lactate metabolism, Wang et al. updates this picture, underscoring the importance of glial-neuron metabolic coupling and lactate transport. Specifically, astrocyte-neuron lactate shuttling is a key mechanism in which astrocytes produce lactate, which neurons take up, a process that is impaired in AD. The review thus suggests that targeting glial lactate metabolism could offer novel approaches for AD diagnosis and treatment. This is complemented by the original research article on dysregulated glucose metabolism in the visual cortex, Mjaaseth et al., in patients with mild cognitive impairment and AD, showing that even in the visual cortex, which is less affected structurally in early AD, metabolic alterations are detectable at early disease stages. This supports the relevance of energy metabolism in AD and suggests that strategies aimed at improving neuronal energy metabolism warrant further investigation.

## Computational biology: for the study of metabolism in neurodegenerative diseases

The collection also reflects the growing role of computational biology. Using bioinformatics, transcriptomics, and machine learning, Noor et al. identified *AKT1* and *PPARGC1A* as candidate diagnostic and therapeutic targets in AD and screened small molecules against these targets. Such approaches may contribute to precision medicine by identifying candidate molecular targets and patient subgroups. Comparable precision approaches have been proposed in PD (Arias-Carrión, 2026). In this context, precision metabolic medicine would stratify patients by metabolic phenotype (for example, obesity, insulin resistance, or diabetes), neurological disease stage, and molecular biomarkers, and test whether these features predict treatment response. Precision metabolomics may contribute by identifying metabolic signatures associated with disease stage or treatment response (Quintero Escobar et al., 2021). These approaches remain to be prospectively validated before clinical use.

Taken together, these contributions support a view of metabolic dysfunction as a potential contributor to neurodegenerative processes, rather than simply a downstream consequence. Its effects may involve modulation of inflammation, oxidative stress, mitochondrial dysfunction, and vascular impairment. However, directionality, timing and causality remain incompletely resolved and are likely to differ by disease and disease stage.

Since this Research Topic was launched, metabolic pathways have moved from epidemiological associations to active therapeutic targets. GLP-1 receptor agonists, developed to improve glycaemic control, have increasingly been shown to modulate brain pathways involved in appetite, energy homeostasis, neuroinflammation and neuronal function (Iwai et al., 2014). Large observational studies have associated their use with reduced dementia incidence (Wang et al., 2025). Recent preclinical work further supports broader effects of GLP-1R activation on aging: semaglutide improved metabolic and physiological function and spatial memory in aged female mice (Feng et al., 2026). However, in the phase 3 EVOKE and EVOKE+ trials, oral semaglutide did not slow clinical decline in early symptomatic AD over 104 weeks on the primary Clinical Dementia Rating–Sum of Boxes (CDR-SB) endpoint, despite changes in several CSF biomarkers (Cummings et al., 2026).

The EVOKE results highlight an important distinction between biological effects and clinical benefit: biomarker changes do not necessarily translate into measurable clinical benefits. Whether earlier intervention, metabolic stratification, different dosing or combination therapies could improve clinical outcomes remains to be tested. Because participants were not stratified by metabolic phenotype, the trials cannot determine whether patients with obesity, insulin resistance or diabetes might respond differently.

The long-term neurological effects of chronically modulating these pathways also remain incompletely understood. Pharmacovigilance data have raised, and only partly resolved, questions about neuropsychiatric effects of GLP-1 receptor agonists, including signals of depression and suicidality alongside evidence of antidepressant potential (Sa et al., 2026). Future studies should therefore consider metabolic and neurological comorbidities, timing of intervention and longer follow-up.

This Research Topic captures a field in transformation. The studies collected here illustrate how altered glucose metabolism, insulin signaling, oxidative stress, glial biology, and vascular dysfunction intersected with neurodegenerative processes, while reinforcing brain metabolism as a potential therapeutic target (Figure 1). The challenge ahead will be to determine not only how these pathways can be modulated, but when, in whom, and for how long. Answering them will require integrating metabolic phenotype, disease stage and biological markers with neuroscience and vascular biology. We hope this collection stimulates further work at this interface.

**Figure 1:**
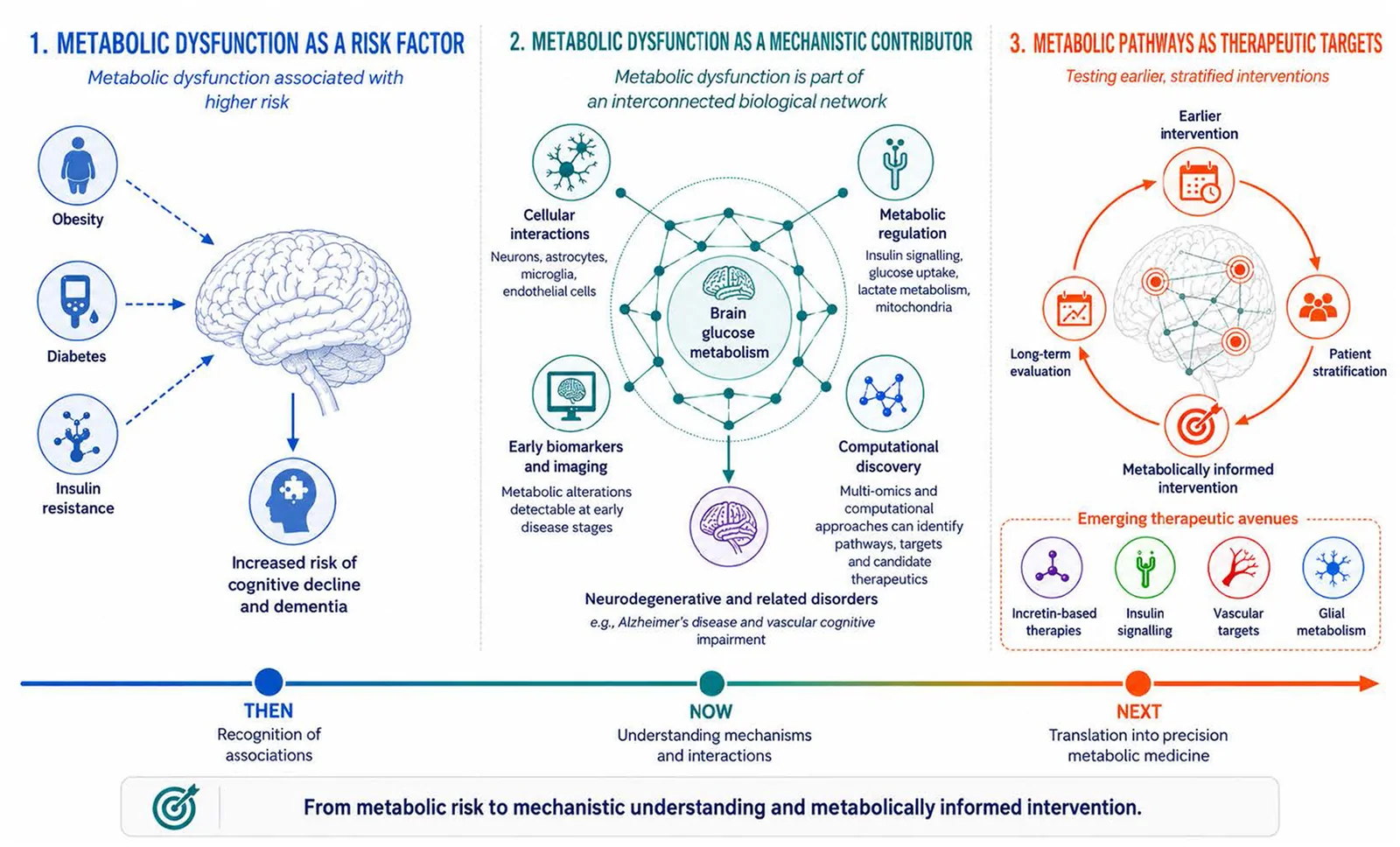
From metabolic risk to precision metabolic medicine in neurodegenerative disease. Schematic representation of the evolving understanding of metabolic dysfunction in neurodegeneration, from epidemiological associations with obesity and diabetes to candidate mechanistic pathways involving insulin resistance, altered glucose metabolism, neuroinflammation, oxidative stress and vascular dysfunction, and ultimately toward metabolically informed therapeutic strategies.
